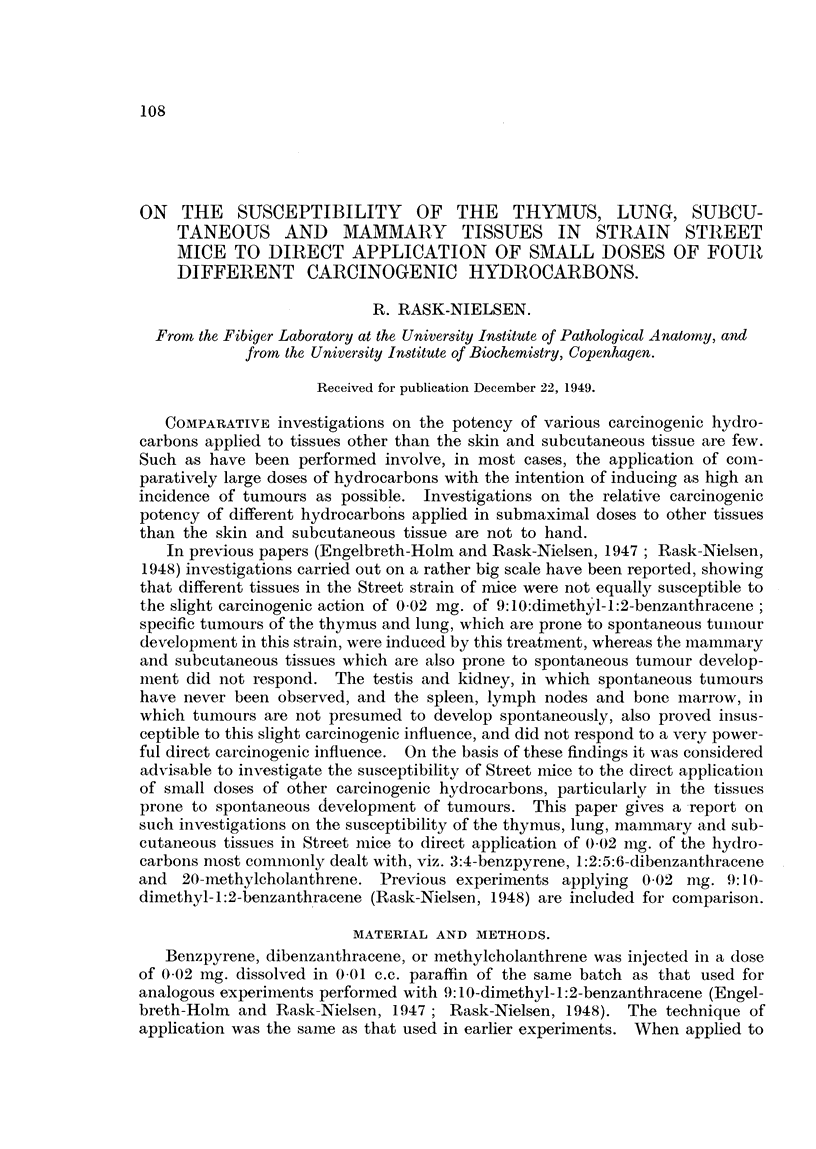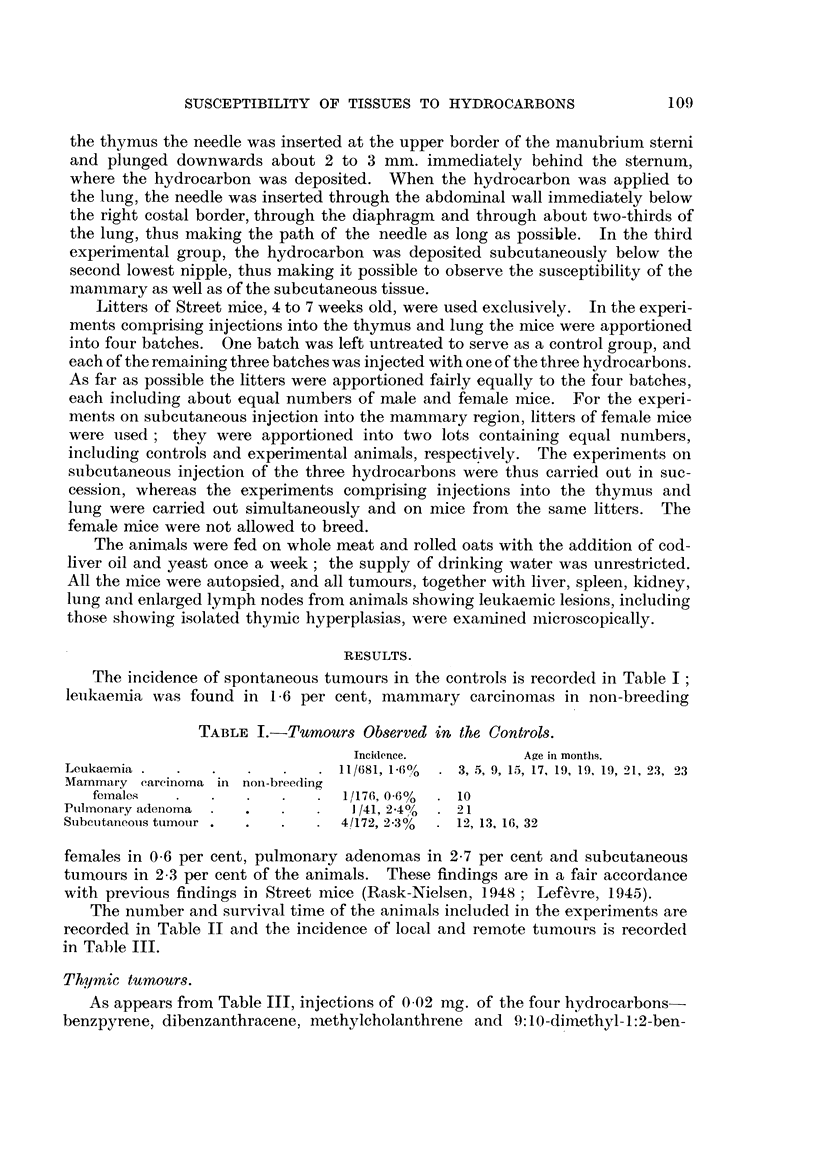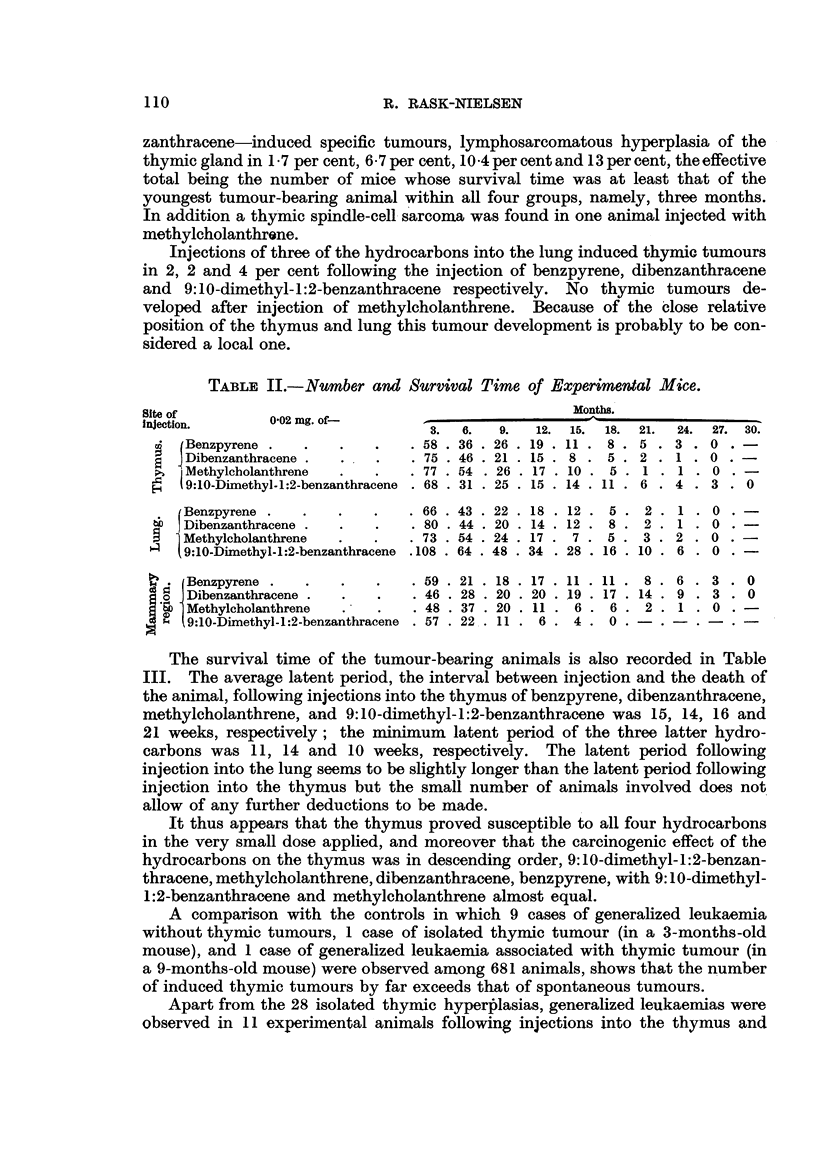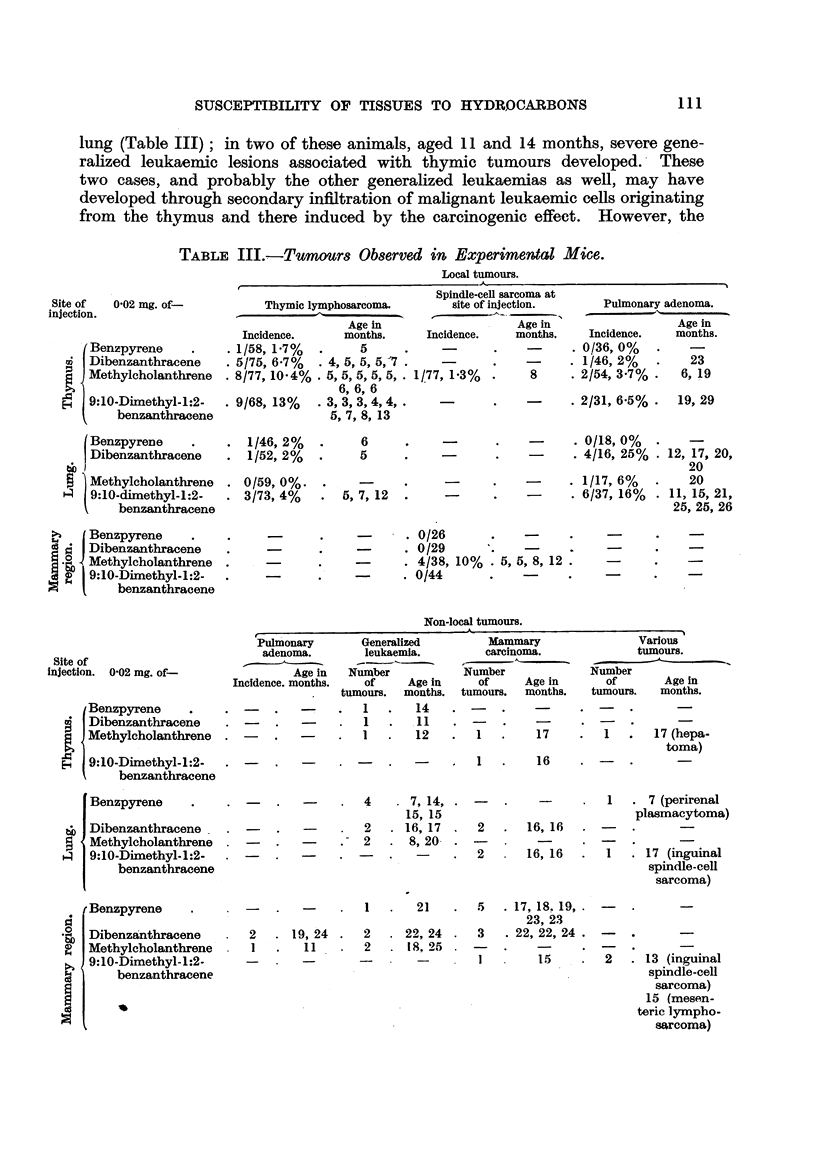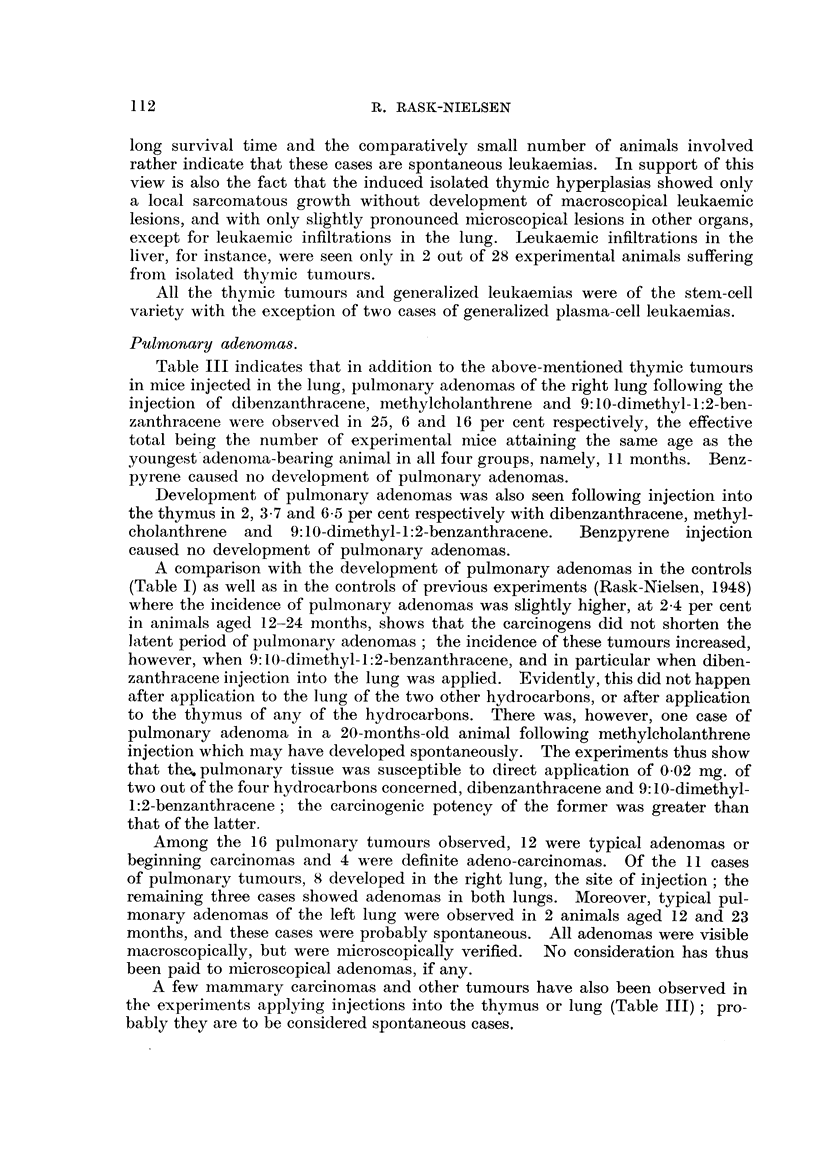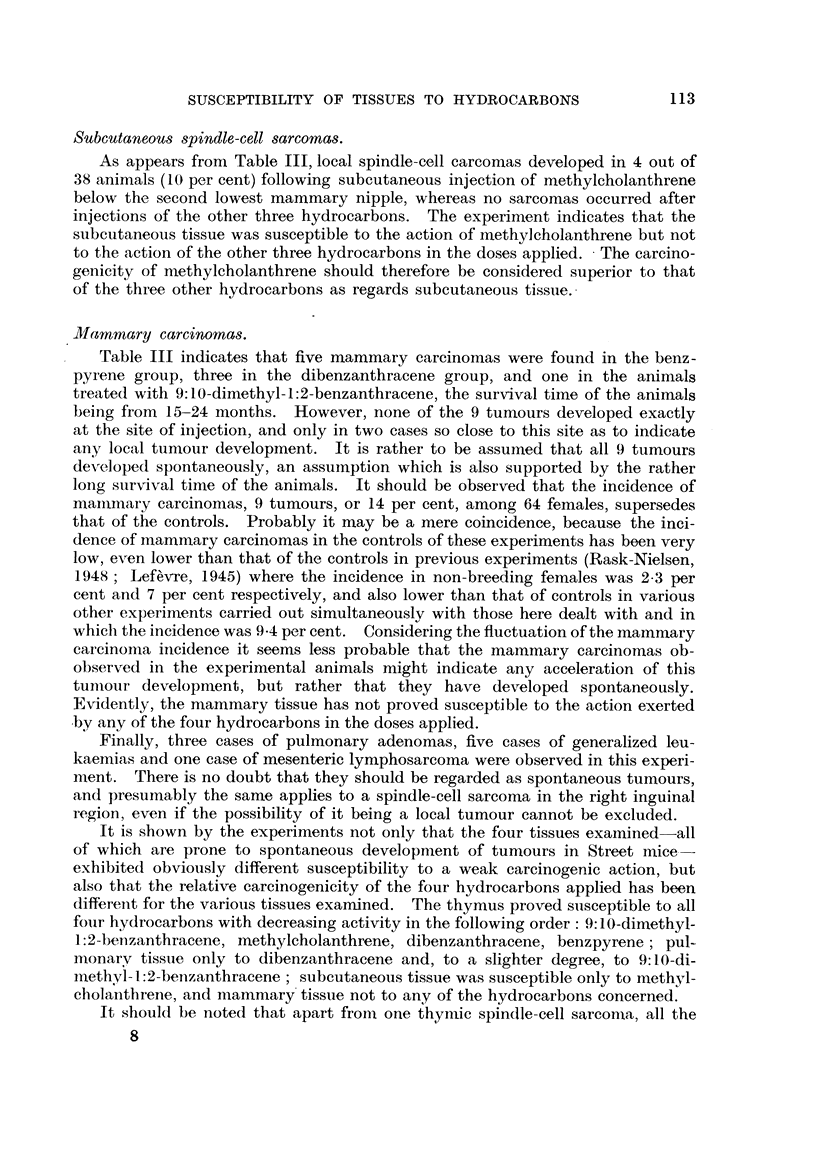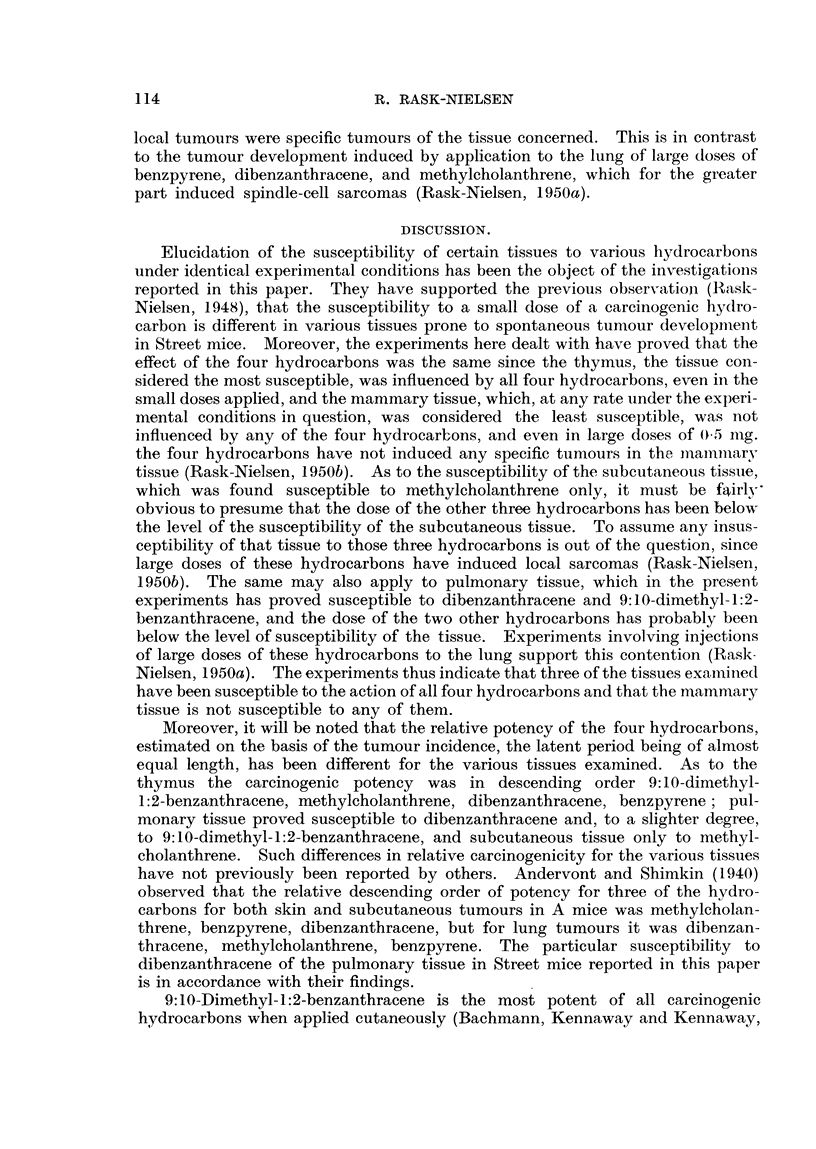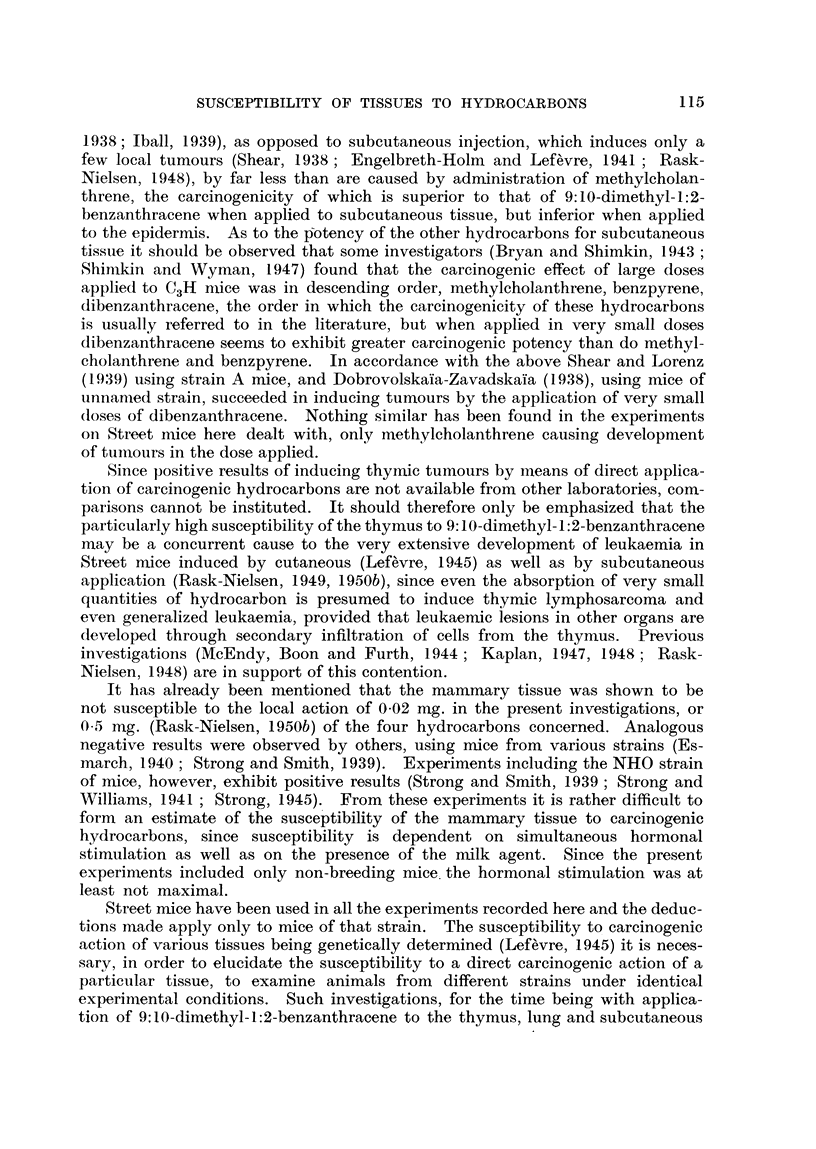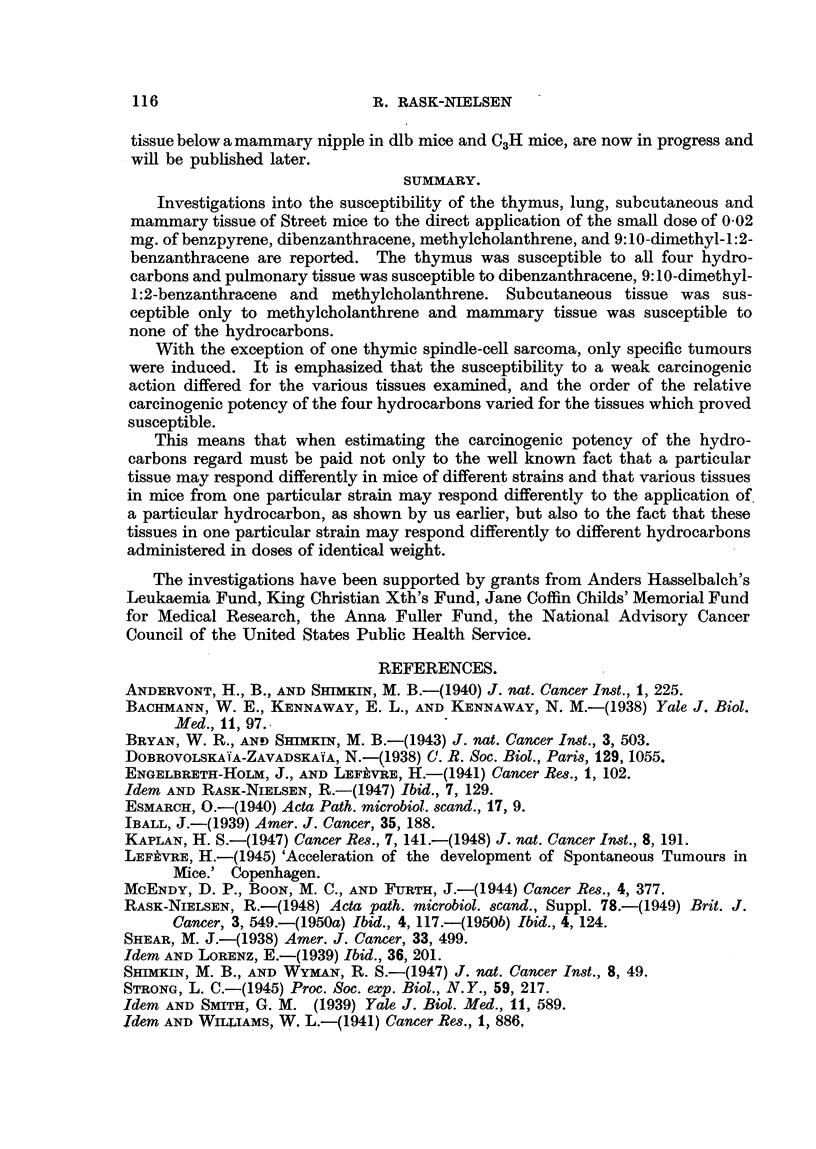# On the Susceptibility of the Thymus, Lung, Subcutaneous and Mammary Tissues in Strain Street Mice to Direct Application of Small Doses of Four Different Carcinogenic Hydrocarbons

**DOI:** 10.1038/bjc.1950.10

**Published:** 1950-03

**Authors:** R. Rask-Nielsen


### ON THE SUSCEPTIBILITY OF THE THYMUS, LUNG, SUBCUTANEOUS AND MAMMARY TISSUES IN STRAIN STREET MICE TO DIRECT APPLICATION OF SMALL DOSES OF FOUR DIFFERENT CARCINOGENIC HYDROCARBONS.

R. RASK-NIELSEN.

*From the Fibiger Laboratory at the University Institute of Pathological Anatomy, and from the University Institute of Biochemistry, Copenhagen.*

Received for publication December 22, 1949.

COMPARATIVE investigations on the potency of various carcinogenic hydrocarbons applied to tissues other than the skin and subcutaneous tissue are few. Such as have been performed involve, in most cases, the application of comparatively large doses of hydrocarbons with the intention of inducing as high an incidence of tumours as possible. Investigations on the relative carcinogenic potency of different hydrocarbons applied in submaximal doses to other tissues than the skin and subcutaneous tissue are not to hand.

In previous papers (Engelbreth-Holm and Rask-Nielsen, 1947 ; Rask-Nielsen, 1948) investigations carried out on a rather big scale have been reported, showing that different tissues in the Street strain of mice were not equally susceptible to the slight carcinogenic action of 0·02 mg. of 9:10:dimethyl-1:2-benzanthracene ; specific tumours of the thymus and lung, which are prone to spontaneous tumour development in this strain, were induced by this treatment, whereas the mammary and subcutaneous tissues which are also prone to spontaneous tumour development did not respond. The testis and kidney, in which spontaneous tumours have never been observed, and the spleen, lymph nodes and bone marrow, in which tumours are not presumed to develop spontaneously, also proved insusceptible to this slight carcinogenic influence, and did not respond to a very powerful direct carcinogenic influence. On the basis of these findings it was considered advisable to investigate the susceptibility of Street mice to the direct application of small doses of other carcinogenic hydrocarbons, particularly in the tissues prone to spontaneous development of tumours. This paper gives a report on such investigations on the susceptibility of the thymus, lung, mammary and subcutaneous tissues in Street mice to direct application of 0·02 mg. of the hydrocarbons most commonly dealt with, viz. 3:4-benzpyrene, 1:2:5:6-dibenzanthracene and 20-methylcholanthrene. Previous experiments applying 0·02 mg. 9:10-dimethyl-1:2-benzanthracene (Rask-Nielsen, 1948) are included for comparison.

### MATERIAL AND METHODS.

Benzpyrene, dibenzanthracene, or methylcholanthrene was injected in a dose of 0·02 mg. dissolved in 0·01 c.c. paraffin of the same batch as that used for analogous experiments performed with 9:10-dimethyl-1:2-benzanthracene (Engelbreth-Holm and Rask-Nielsen, 1947 ; Rask-Nielsen, 1948). The technique of application was the same as that used in earlier experiments. When applied to the thymus the needle was inserted at the upper border of the manubrium sterni and plunged downwards about 2 to 3 mm. immediately behind the sternum, where the hydrocarbon was deposited. When the hydrocarbon was applied to the lung, the needle was inserted through the abdominal wall immediately below the right costal border, through the diaphragm and through about two-thirds of the lung, thus making the path of the needle as long as possible. In the third experimental group, the hydrocarbon was deposited subcutaneously below the second lowest nipple, thus making it possible to observe the susceptibility of the mammary as well as of the subcutaneous tissue.

Litters of Street mice, 4 to 7 weeks old, were used exclusively. In the experiments comprising injections into the thymus and lung the mice were apportioned into four batches. One batch was left untreated to serve as a control group, and each of the remaining three batches was injected with one of the three hydrocarbons. As far as possible the litters were apportioned fairly equally to the four batches, each including about equal numbers of male and female mice. For the experiments on subcutaneous injection into the mammary region, litters of female mice were used ; they were apportioned into two lots containing equal numbers, including controls and experimental animals, respectively. The experiments on subcutaneous injection of the three hydrocarbons were thus carried out in succession, whereas the experiments comprising injections into the thymus and lung were carried out simultaneously and on mice from the same litters. The female mice were not allowed to breed.

The animals were fed on whole meat and rolled oats with the addition of cod-liver oil and yeast once a week ; the supply of drinking water was unrestricted. All the mice were autopsied, and all tumours, together with liver, spleen, kidney, lung and enlarged lymph nodes from animals showing leukaemic lesions, including those showing isolated thymic hyperplasias, were examined microscopically.

### RESULTS.

The incidence of spontaneous tumours in the controls is recorded in Table I ; leukaemia was found in 1·6 per cent, mammary carcinomas in non-breeding

TABLE I.—*Tumours Observed in the Controls.*

| | Incidence. | Age in months. |
|---|---|---|
| Leukaemia | 11/681, 1·6% | 3, 5, 9, 15, 17, 19, 19, 19, 21, 23, 23 |
| Mammary carcinoma in non-breeding females | 1/176, 0·6% | 10 |
| Pulmonary adenoma | 1/41, 2·4% | 21 |
| Subcutaneous tumour | 4/172, 2·3% | 12, 13, 16, 32 |

females in 0·6 per cent, pulmonary adenomas in 2·7 per cent and subcutaneous tumours in 2·3 per cent of the animals. These findings are in a fair accordance with previous findings in Street mice (Rask-Nielsen, 1948 ; Lefèvre, 1945).

The number and survival time of the animals included in the experiments are recorded in Table II and the incidence of local and remote tumours is recorded in Table III.

*Thymic tumours.*

As appears from Table III, injections of 0·02 mg. of the four hydrocarbons—benzpyrene, dibenzanthracene, methylcholanthrene and 9:10-dimethyl-1:2-benzanthracene—induced specific tumours, lymphosarcomatous hyperplasia of the thymic gland in 1·7 per cent, 6·7 per cent, 10·4 per cent and 13 per cent, the effective total being the number of mice whose survival time was at least that of the youngest tumour-bearing animal within all four groups, namely, three months. In addition a thymic spindle-cell sarcoma was found in one animal injected with methylcholanthrene.

Injections of three of the hydrocarbons into the lung induced thymic tumours in 2, 2 and 4 per cent following the injection of benzpyrene, dibenzanthracene and 9:10-dimethyl-1:2-benzanthracene respectively. No thymic tumours developed after injection of methylcholanthrene. Because of the close relative position of the thymus and lung this tumour development is probably to be considered a local one.

TABLE II.—*Number and Survival Time of Experimental Mice.*

| Site of injection. | 0·02 mg. of— | Months. 3. | 6. | 9. | 12. | 15. | 18. | 21. | 24. | 27. | 30. |
|---|---|---|---|---|---|---|---|---|---|---|---|
| Thymus. | Benzpyrene | 58 | 36 | 26 | 19 | 11 | 8 | 5 | 3 | 0 | — |
| | Dibenzanthracene | 75 | 46 | 21 | 15 | 8 | 5 | 2 | 1 | 0 | — |
| | Methylcholanthrene | 77 | 54 | 26 | 17 | 10 | 5 | 1 | 1 | 0 | — |
| | 9:10-Dimethyl-1:2-benzanthracene | 68 | 31 | 25 | 15 | 14 | 11 | 6 | 4 | 3 | 0 |
| Lung. | Benzpyrene | 66 | 43 | 22 | 18 | 12 | 5 | 2 | 1 | 0 | — |
| | Dibenzanthracene | 80 | 44 | 20 | 14 | 12 | 8 | 2 | 1 | 0 | — |
| | Methylcholanthrene | 73 | 54 | 24 | 17 | 7 | 5 | 3 | 2 | 0 | — |
| | 9:10-Dimethyl-1:2-benzanthracene | 108 | 64 | 48 | 34 | 28 | 16 | 10 | 6 | 0 | — |
| Mammary region. | Benzpyrene | 59 | 21 | 18 | 17 | 11 | 11 | 8 | 6 | 3 | 0 |
| | Dibenzanthracene | 46 | 28 | 20 | 20 | 19 | 17 | 14 | 9 | 3 | 0 |
| | Methylcholanthrene | 48 | 37 | 20 | 11 | 6 | 6 | 2 | 1 | 0 | — |
| | 9:10-Dimethyl-1:2-benzanthracene | 57 | 22 | 11 | 6 | 4 | 0 | — | — | — | — |

The survival time of the tumour-bearing animals is also recorded in Table III. The average latent period, the interval between injection and the death of the animal, following injections into the thymus of benzpyrene, dibenzanthracene, methylcholanthrene, and 9:10-dimethyl-1:2-benzanthracene was 15, 14, 16 and 21 weeks, respectively ; the minimum latent period of the three latter hydrocarbons was 11, 14 and 10 weeks, respectively. The latent period following injection into the lung seems to be slightly longer than the latent period following injection into the thymus but the small number of animals involved does not allow of any further deductions to be made.

It thus appears that the thymus proved susceptible to all four hydrocarbons in the very small dose applied, and moreover that the carcinogenic effect of the hydrocarbons on the thymus was in descending order, 9:10-dimethyl-1:2-benzanthracene, methylcholanthrene, dibenzanthracene, benzpyrene, with 9:10-dimethyl-1:2-benzanthracene and methylcholanthrene almost equal.

A comparison with the controls in which 9 cases of generalized leukaemia without thymic tumours, 1 case of isolated thymic tumour (in a 3-months-old mouse), and 1 case of generalized leukaemia associated with thymic tumour (in a 9-months-old mouse) were observed among 681 animals, shows that the number of induced thymic tumours by far exceeds that of spontaneous tumours.

Apart from the 28 isolated thymic hyperplasias, generalized leukaemias were observed in 11 experimental animals following injections into the thymus and lung (Table III); in two of these animals, aged 11 and 14 months, severe generalized leukaemic lesions associated with thymic tumours developed. These two cases, and probably the other generalized leukaemias as well, may have developed through secondary infiltration of malignant leukaemic cells originating from the thymus and there induced by the carcinogenic effect. However, the

TABLE III.—*Tumours Observed in Experimental Mice.*

Local tumours.

| Site of injection. | 0·02 mg. of— | Thymic lymphosarcoma. Incidence. | Thymic lymphosarcoma. Age in months. | Spindle-cell sarcoma at site of injection. Incidence. | Spindle-cell sarcoma at site of injection. Age in months. | Pulmonary adenoma. Incidence. | Pulmonary adenoma. Age in months. |
|---|---|---|---|---|---|---|---|
| Thymus. | Benzpyrene | 1/58, 1·7% | 5 | — | — | 0/36, 0% | — |
| | Dibenzanthracene | 5/75, 6·7% | 4, 5, 5, 5, 7 | — | — | 1/46, 2% | 23 |
| | Methylcholanthrene | 8/77, 10·4% | 5, 5, 5, 5, 5, 6, 6, 6 | 1/77, 1·3% | 8 | 2/54, 3·7% | 6, 19 |
| | 9:10-Dimethyl-1:2-benzanthracene | 9/68, 13% | 3, 3, 3, 4, 4, 5, 7, 8, 13 | — | — | 2/31, 6·5% | 19, 29 |
| Lung. | Benzpyrene | 1/46, 2% | 6 | — | — | 0/18, 0% | — |
| | Dibenzanthracene | 1/52, 2% | 5 | — | — | 4/16, 25% | 12, 17, 20, 20 |
| | Methylcholanthrene | 0/59, 0% | — | — | — | 1/17, 6% | 20 |
| | 9:10-dimethyl-1:2-benzanthracene | 3/73, 4% | 5, 7, 12 | — | — | 6/37, 16% | 11, 15, 21, 25, 25, 26 |
| Mammary region. | Benzpyrene | — | — | 0/26 | — | — | — |
| | Dibenzanthracene | — | — | 0/29 | — | — | — |
| | Methylcholanthrene | — | — | 4/38, 10% | 5, 5, 8, 12 | — | — |
| | 9:10-Dimethyl-1:2-benzanthracene | — | — | 0/44 | — | — | — |

Non-local tumours.

| Site of injection. | 0·02 mg. of— | Pulmonary adenoma. Incidence. | Pulmonary adenoma. Age in months. | Generalized leukaemia. Number of tumours. | Generalized leukaemia. Age in months. | Mammary carcinoma. Number of tumours. | Mammary carcinoma. Age in months. | Various tumours. Number of tumours. | Various tumours. Age in months. |
|---|---|---|---|---|---|---|---|---|---|
| Thymus. | Benzpyrene | — | — | 1 | 14 | — | — | — | — |
| | Dibenzanthracene | — | — | 1 | 11 | — | — | — | — |
| | Methylcholanthrene | — | — | 1 | 12 | 1 | 17 | 1 | 17 (hepatoma) |
| | 9:10-Dimethyl-1:2-benzanthracene | — | — | — | — | 1 | 16 | — | — |
| Lung. | Benzpyrene | — | — | 4 | 7, 14, 15, 15 | — | — | 1 | 7 (perirenal plasmacytoma) |
| | Dibenzanthracene | — | — | 2 | 16, 17 | 2 | 16, 16 | — | — |
| | Methylcholanthrene | — | — | 2 | 8, 20 | — | — | — | — |
| | 9:10-Dimethyl-1:2-benzanthracene | — | — | — | — | 2 | 16, 16 | 1 | 17 (inguinal spindle-cell sarcoma) |
| Mammary region. | Benzpyrene | — | — | 1 | 21 | 5 | 17, 18, 19, 23, 23 | — | — |
| | Dibenzanthracene | 2 | 19, 24 | 2 | 22, 24 | 3 | 22, 22, 24 | — | — |
| | Methylcholanthrene | 1 | 11 | 2 | 18, 25 | — | — | — | — |
| | 9:10-Dimethyl-1:2-benzanthracene | — | — | — | — | 1 | 15 | 2 | 13 (inguinal spindle-cell sarcoma) 15 (mesenteric lymphosarcoma) |

long survival time and the comparatively small number of animals involved rather indicate that these cases are spontaneous leukaemias. In support of this view is also the fact that the induced isolated thymic hyperplasias showed only a local sarcomatous growth without development of macroscopical leukaemic lesions, and with only slightly pronounced microscopical lesions in other organs, except for leukaemic infiltrations in the lung. Leukaemic infiltrations in the liver, for instance, were seen only in 2 out of 28 experimental animals suffering from isolated thymic tumours.

All the thymic tumours and generalized leukaemias were of the stem-cell variety with the exception of two cases of generalized plasma-cell leukaemias.

*Pulmonary adenomas.*

Table III indicates that in addition to the above-mentioned thymic tumours in mice injected in the lung, pulmonary adenomas of the right lung following the injection of dibenzanthracene, methylcholanthrene and 9:10-dimethyl-1:2-benzanthracene were observed in 25, 6 and 16 per cent respectively, the effective total being the number of experimental mice attaining the same age as the youngest adenoma-bearing animal in all four groups, namely, 11 months. Benzpyrene caused no development of pulmonary adenomas.

Development of pulmonary adenomas was also seen following injection into the thymus in 2, 3·7 and 6·5 per cent respectively with dibenzanthracene, methylcholanthrene and 9:10-dimethyl-1:2-benzanthracene. Benzpyrene injection caused no development of pulmonary adenomas.

A comparison with the development of pulmonary adenomas in the controls (Table I) as well as in the controls of previous experiments (Rask-Nielsen, 1948) where the incidence of pulmonary adenomas was slightly higher, at 2·4 per cent in animals aged 12–24 months, shows that the carcinogens did not shorten the latent period of pulmonary adenomas ; the incidence of these tumours increased, however, when 9:10-dimethyl-1:2-benzanthracene, and in particular when dibenzanthracene injection into the lung was applied. Evidently, this did not happen after application to the lung of the two other hydrocarbons, or after application to the thymus of any of the hydrocarbons. There was, however, one case of pulmonary adenoma in a 20-months-old animal following methylcholanthrene injection which may have developed spontaneously. The experiments thus show that the pulmonary tissue was susceptible to direct application of 0·02 mg. of two out of the four hydrocarbons concerned, dibenzanthracene and 9:10-dimethyl-1:2-benzanthracene ; the carcinogenic potency of the former was greater than that of the latter.

Among the 16 pulmonary tumours observed, 12 were typical adenomas or beginning carcinomas and 4 were definite adeno-carcinomas. Of the 11 cases of pulmonary tumours, 8 developed in the right lung, the site of injection ; the remaining three cases showed adenomas in both lungs. Moreover, typical pulmonary adenomas of the left lung were observed in 2 animals aged 12 and 23 months, and these cases were probably spontaneous. All adenomas were visible macroscopically, but were microscopically verified. No consideration has thus been paid to microscopical adenomas, if any.

A few mammary carcinomas and other tumours have also been observed in the experiments applying injections into the thymus or lung (Table III) ; probably they are to be considered spontaneous cases.

*Subcutaneous spindle-cell sarcomas.*

As appears from Table III, local spindle-cell carcomas developed in 4 out of 38 animals (10 per cent) following subcutaneous injection of methylcholanthrene below the second lowest mammary nipple, whereas no sarcomas occurred after injections of the other three hydrocarbons. The experiment indicates that the subcutaneous tissue was susceptible to the action of methylcholanthrene but not to the action of the other three hydrocarbons in the doses applied. The carcinogenicity of methylcholanthrene should therefore be considered superior to that of the three other hydrocarbons as regards subcutaneous tissue.

*Mammary carcinomas.*

Table III indicates that five mammary carcinomas were found in the benzpyrene group, three in the dibenzanthracene group, and one in the animals treated with 9:10-dimethyl-1:2-benzanthracene, the survival time of the animals being from 15–24 months. However, none of the 9 tumours developed exactly at the site of injection, and only in two cases so close to this site as to indicate any local tumour development. It is rather to be assumed that all 9 tumours developed spontaneously, an assumption which is also supported by the rather long survival time of the animals. It should be observed that the incidence of mammary carcinomas, 9 tumours, or 14 per cent, among 64 females, supersedes that of the controls. Probably it may be a mere coincidence, because the incidence of mammary carcinomas in the controls of these experiments has been very low, even lower than that of the controls in previous experiments (Rask-Nielsen, 1948; Lefèvre, 1945) where the incidence in non-breeding females was 2·3 per cent and 7 per cent respectively, and also lower than that of controls in various other experiments carried out simultaneously with those here dealt with and in which the incidence was 9·4 per cent. Considering the fluctuation of the mammary carcinoma incidence it seems less probable that the mammary carcinomas obobserved in the experimental animals might indicate any acceleration of this tumour development, but rather that they have developed spontaneously. Evidently, the mammary tissue has not proved susceptible to the action exerted by any of the four hydrocarbons in the doses applied.

Finally, three cases of pulmonary adenomas, five cases of generalized leukaemias and one case of mesenteric lymphosarcoma were observed in this experiment. There is no doubt that they should be regarded as spontaneous tumours, and presumably the same applies to a spindle-cell sarcoma in the right inguinal region, even if the possibility of it being a local tumour cannot be excluded.

It is shown by the experiments not only that the four tissues examined—all of which are prone to spontaneous development of tumours in Street mice—exhibited obviously different susceptibility to a weak carcinogenic action, but also that the relative carcinogenicity of the four hydrocarbons applied has been different for the various tissues examined. The thymus proved susceptible to all four hydrocarbons with decreasing activity in the following order: 9:10-dimethyl-1:2-benzanthracene, methylcholanthrene, dibenzanthracene, benzpyrene; pulmonary tissue only to dibenzanthracene and, to a slighter degree, to 9:10-dimethyl-1:2-benzanthracene; subcutaneous tissue was susceptible only to methylcholanthrene, and mammary tissue not to any of the hydrocarbons concerned.

It should be noted that apart from one thymic spindle-cell sarcoma, all the local tumours were specific tumours of the tissue concerned. This is in contrast to the tumour development induced by application to the lung of large doses of benzpyrene, dibenzanthracene, and methylcholanthrene, which for the greater part induced spindle-cell sarcomas (Rask-Nielsen, 1950*a*).

### DISCUSSION.

Elucidation of the susceptibility of certain tissues to various hydrocarbons under identical experimental conditions has been the object of the investigations reported in this paper. They have supported the previous observation (Rask-Nielsen, 1948), that the susceptibility to a small dose of a carcinogenic hydrocarbon is different in various tissues prone to spontaneous tumour development in Street mice. Moreover, the experiments here dealt with have proved that the effect of the four hydrocarbons was the same since the thymus, the tissue considered the most susceptible, was influenced by all four hydrocarbons, even in the small doses applied, and the mammary tissue, which, at any rate under the experimental conditions in question, was considered the least susceptible, was not influenced by any of the four hydrocarbons, and even in large doses of 0·5 mg. the four hydrocarbons have not induced any specific tumours in the mammary tissue (Rask-Nielsen, 1950*b*). As to the susceptibility of the subcutaneous tissue, which was found susceptible to methylcholanthrene only, it must be fairly obvious to presume that the dose of the other three hydrocarbons has been below the level of the susceptibility of the subcutaneous tissue. To assume any insusceptibility of that tissue to those three hydrocarbons is out of the question, since large doses of these hydrocarbons have induced local sarcomas (Rask-Nielsen, 1950*b*). The same may also apply to pulmonary tissue, which in the present experiments has proved susceptible to dibenzanthracene and 9:10-dimethyl-1:2-benzanthracene, and the dose of the two other hydrocarbons has probably been below the level of susceptibility of the tissue. Experiments involving injections of large doses of these hydrocarbons to the lung support this contention (Rask-Nielsen, 1950*a*). The experiments thus indicate that three of the tissues examined have been susceptible to the action of all four hydrocarbons and that the mammary tissue is not susceptible to any of them.

Moreover, it will be noted that the relative potency of the four hydrocarbons, estimated on the basis of the tumour incidence, the latent period being of almost equal length, has been different for the various tissues examined. As to the thymus the carcinogenic potency was in descending order 9:10-dimethyl-1:2-benzanthracene, methylcholanthrene, dibenzanthracene, benzpyrene; pulmonary tissue proved susceptible to dibenzanthracene and, to a slighter degree, to 9:10-dimethyl-1:2-benzanthracene, and subcutaneous tissue only to methylcholanthrene. Such differences in relative carcinogenicity for the various tissues have not previously been reported by others. Andervont and Shimkin (1940) observed that the relative descending order of potency for three of the hydrocarbons for both skin and subcutaneous tumours in A mice was methylcholanthrene, benzpyrene, dibenzanthracene, but for lung tumours it was dibenzanthracene, methylcholanthrene, benzpyrene. The particular susceptibility to dibenzanthracene of the pulmonary tissue in Street mice reported in this paper is in accordance with their findings.

9:10-Dimethyl-1:2-benzanthracene is the most potent of all carcinogenic hydrocarbons when applied cutaneously (Bachmann, Kennaway and Kennaway,

1938; Iball, 1939), as opposed to subcutaneous injection, which induces only a few local tumours (Shear, 1938; Engelbreth-Holm and Lefèvre, 1941; Rask-Nielsen, 1948), by far less than are caused by administration of methylcholanthrene, the carcinogenicity of which is superior to that of 9:10-dimethyl-1:2-benzanthracene when applied to subcutaneous tissue, but inferior when applied to the epidermis. As to the potency of the other hydrocarbons for subcutaneous tissue it should be observed that some investigators (Bryan and Shimkin, 1943; Shimkin and Wyman, 1947) found that the carcinogenic effect of large doses applied to $C_3H$ mice was in descending order, methylcholanthrene, benzpyrene, dibenzanthracene, the order in which the carcinogenicity of these hydrocarbons is usually referred to in the literature, but when applied in very small doses dibenzanthracene seems to exhibit greater carcinogenic potency than do methylcholanthrene and benzpyrene. In accordance with the above Shear and Lorenz (1939) using strain A mice, and Dobrovolskaïa-Zavadskaïa (1938), using mice of unnamed strain, succeeded in inducing tumours by the application of very small doses of dibenzanthracene. Nothing similar has been found in the experiments on Street mice here dealt with, only methylcholanthrene causing development of tumours in the dose applied.

Since positive results of inducing thymic tumours by means of direct application of carcinogenic hydrocarbons are not available from other laboratories, comparisons cannot be instituted. It should therefore only be emphasized that the particularly high susceptibility of the thymus to 9:10-dimethyl-1:2-benzanthracene may be a concurrent cause to the very extensive development of leukaemia in Street mice induced by cutaneous (Lefèvre, 1945) as well as by subcutaneous application (Rask-Nielsen, 1949, 1950*b*), since even the absorption of very small quantities of hydrocarbon is presumed to induce thymic lymphosarcoma and even generalized leukaemia, provided that leukaemic lesions in other organs are developed through secondary infiltration of cells from the thymus. Previous investigations (McEndy, Boon and Furth, 1944; Kaplan, 1947, 1948; Rask-Nielsen, 1948) are in support of this contention.

It has already been mentioned that the mammary tissue was shown to be not susceptible to the local action of 0·02 mg. in the present investigations, or 0·5 mg. (Rask-Nielsen, 1950*b*) of the four hydrocarbons concerned. Analogous negative results were observed by others, using mice from various strains (Esmarch, 1940; Strong and Smith, 1939). Experiments including the NHO strain of mice, however, exhibit positive results (Strong and Smith, 1939; Strong and Williams, 1941; Strong, 1945). From these experiments it is rather difficult to form an estimate of the susceptibility of the mammary tissue to carcinogenic hydrocarbons, since susceptibility is dependent on simultaneous hormonal stimulation as well as on the presence of the milk agent. Since the present experiments included only non-breeding mice. the hormonal stimulation was at least not maximal.

Street mice have been used in all the experiments recorded here and the deductions made apply only to mice of that strain. The susceptibility to carcinogenic action of various tissues being genetically determined (Lefèvre, 1945) it is necessary, in order to elucidate the susceptibility to a direct carcinogenic action of a particular tissue, to examine animals from different strains under identical experimental conditions. Such investigations, for the time being with application of 9:10-dimethyl-1:2-benzanthracene to the thymus, lung and subcutaneous tissue below a mammary nipple in dlb mice and $C_3H$ mice, are now in progress and will be published later.

SUMMARY.

Investigations into the susceptibility of the thymus, lung, subcutaneous and mammary tissue of Street mice to the direct application of the small dose of 0·02 mg. of benzpyrene, dibenzanthracene, methylcholanthrene, and 9:10-dimethyl-1:2-benzanthracene are reported. The thymus was susceptible to all four hydrocarbons and pulmonary tissue was susceptible to dibenzanthracene, 9:10-dimethyl-1:2-benzanthracene and methylcholanthrene. Subcutaneous tissue was susceptible only to methylcholanthrene and mammary tissue was susceptible to none of the hydrocarbons.

With the exception of one thymic spindle-cell sarcoma, only specific tumours were induced. It is emphasized that the susceptibility to a weak carcinogenic action differed for the various tissues examined, and the order of the relative carcinogenic potency of the four hydrocarbons varied for the tissues which proved susceptible.

This means that when estimating the carcinogenic potency of the hydrocarbons regard must be paid not only to the well known fact that a particular tissue may respond differently in mice of different strains and that various tissues in mice from one particular strain may respond differently to the application of a particular hydrocarbon, as shown by us earlier, but also to the fact that these tissues in one particular strain may respond differently to different hydrocarbons administered in doses of identical weight.

The investigations have been supported by grants from Anders Hasselbalch's Leukaemia Fund, King Christian Xth's Fund, Jane Coffin Childs' Memorial Fund for Medical Research, the Anna Fuller Fund, the National Advisory Cancer Council of the United States Public Health Service.

REFERENCES.

ANDERVONT, H., B., AND SHIMKIN, M. B.—(1940) *J. nat. Cancer Inst.*, **1**, 225.
BACHMANN, W. E., KENNAWAY, E. L., AND KENNAWAY, N. M.—(1938) *Yale J. Biol. Med.*, **11**, 97.
BRYAN, W. R., AND SHIMKIN, M. B.—(1943) *J. nat. Cancer Inst.*, **3**, 503.
DOBROVOLSKAÏA-ZAVADSKAÏA, N.—(1938) *C. R. Soc. Biol., Paris*, **129**, 1055.
ENGELBRETH-HOLM, J., AND LEFÈVRE, H.—(1941) *Cancer Res.*, **1**, 102.
*Idem* AND RASK-NIELSEN, R.—(1947) *Ibid.*, **7**, 129.
ESMARCH, O.—(1940) *Acta Path. microbiol. scand.*, **17**, 9.
IBALL, J.—(1939) *Amer. J. Cancer*, **35**, 188.
KAPLAN, H. S.—(1947) *Cancer Res.*, **7**, 141.—(1948) *J. nat. Cancer Inst.*, **8**, 191.
LEFÈVRE, H.—(1945) 'Acceleration of the development of Spontaneous Tumours in Mice.' Copenhagen.
MCENDY, D. P., BOON, M. C., AND FURTH, J.—(1944) *Cancer Res.*, **4**, 377.
RASK-NIELSEN, R.—(1948) *Acta path. microbiol. scand.*, Suppl. **78**.—(1949) *Brit. J. Cancer*, **3**, 549.—(1950*a*) *Ibid.*, **4**, 117.—(1950*b*) *Ibid.*, **4**, 124.
SHEAR, M. J.—(1938) *Amer. J. Cancer*, **33**, 499.
*Idem* AND LORENZ, E.—(1939) *Ibid.*, **36**, 201.
SHIMKIN, M. B., AND WYMAN, R. S.—(1947) *J. nat. Cancer Inst.*, **8**, 49.
STRONG, L. C.—(1945) *Proc. Soc. exp. Biol., N.Y.*, **59**, 217.
*Idem* AND SMITH, G. M. (1939) *Yale J. Biol. Med.*, **11**, 589.
*Idem* AND WILLIAMS, W. L.—(1941) *Cancer Res.*, **1**, 886.